# Phylogenetic annotation of *Drosophila melanogaster* heat shock protein 70 genes

**DOI:** 10.17912/micropub.biology.000558

**Published:** 2022-04-20

**Authors:** Gen Kaneko

**Affiliations:** 1 University of Houston-Victoria

## Abstract

The traditional classification of stress-inducible 70 kDa heat shock protein (Hsp70) and heat shock cognate (Hsc70) requires a revision because of a recent finding that neither of them constitutes a monophyletic gene family. Here we inferred a phylogenetic relationship among *Drosophila melanogaster* Hsp70 family members. *D. melanogaster* Hsp70 family members were separated into four known metazoan Hsp70 lineages: cytosolic A, cytosolic B, endoplasmic reticulum, and mitochondria. Hsc70s sporadically distributed in the phylogenetic tree, indicating their paraphyletic origin. Detailed sequence inspection found several motifs that support the phylogenetic analysis. Taken together, we propose new aliases of *D. melanogaster* Hsp70 family members based on their evolutionary history.

**Figure 1. Evolutionary characterization of Drosophila melanogaster 70 kDa heat shock protein (Hsp70) genes.**
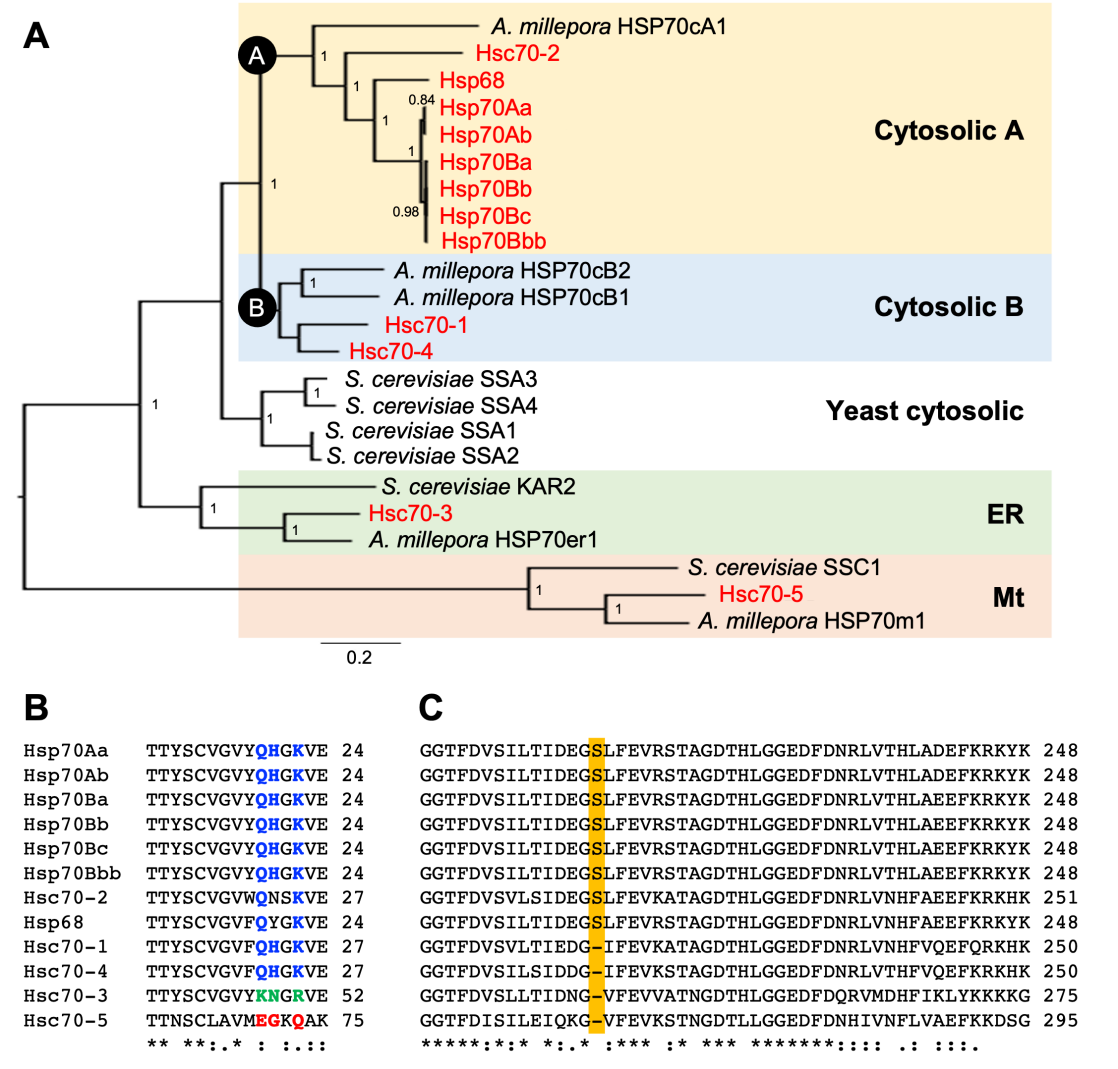
(A) Bayesian phylogenetic tree using the LG+I+G model. Numbers indicate the posterior probability support for each node. Accession numbers for the yeast *Saccharomyces cerevisiae* Hsp70s: SSA1, YAL005C; SSA2, YLL024C; SSA3, YBL075C; SSA4, YER103W; KAR2, YJL034W; SSC1, YJR045C. Accession numbers for the coral *Acropora millepora* Hsp70s: HSP70cA1, XP_029210797.1; HSP70cB1, XP_029206535.1; HSP70cB2, XP_029194855.1; HSP70erA1, XP_029187601.1; HSP70mA1, XP_029208733.1. (B, C) Amino acid sequence alignment of *D. melanogaster* Hsp70s. Numbers on the right margin indicate amino acid numbers from the N-terminus. Residues specific to the cytosolic, endoplasmic reticulum, and mitochondrial lineages are shown in blue, green, and red, respectively. The serine residue specific to the cytosolic A lineage is highlighted.

## Description

The 70 kDa heat shock proteins (Hsp70s) constitute an important family of molecular chaperones. The Hsp70 family members have been classified into stress-inducible Hsp70s and constitutive heat shock cognates (Hsc70s), but this traditional nomenclature warrants revision from the following reasons. First, neither metazoan Hsp70 nor Hsc70 is monophyletic (Yu et al. 2021). This fact also indicates that stress inducibility of metazoan Hsp70s has evolved in several different lineages as also reported in *Paramecium* (Krenek et al. 2013), and the classification based on such a heterogenous feature does not have a strong scientific foundation. Second, it is difficult to explicitly define “stress-inducible” and “constitutive” because the expression of the Hsp70 family members is regulated by various mechanisms including the traditional stress response and unfolded protein response (Garbuz 2017). Third, this traditional nomenclature is causing a serious confusion in Hsp70 studies. Namely, newly identified Hsp70 family member genes are annotated as “ *hsc70* ” when they show a high sequence similarity to known Hsc70 genes. However, the newly identified “Hsc70” genes are not necessarily constitutive because of the possible multiple acquisitions of heat inducibility described above. In the first place, expression pattern is in principle determined by the promoter sequence, not by the coding sequence, and it is inappropriate to confer a name including expression information upon the similarity of the coding sequence. The accelerating accumulation of genome sequences and automatic annotation have yielded many predicted “Hsc70” genes with no expression information, and several “stress-inducible Hsc70s” have even been reported from arthropods and mollusks (Chuang et al. 2007; Li et al. 2018).

Human HSP70s have solved these problems by introducing a new nomenclature (HSPA1, HSPA2, etc.), in which gene names no longer include the expression information (Kampinga et al. 2009). This nomenclature, however, cannot be directly applied to Hsp70s from other organisms because many human Hsp70s likely resulted from duplications specific to the phylum Chordata and subphylum Craniata (Wada et al., 2006). We recently proposed a new nomenclature for metazoan Hsp70s based on the evolutionary history, which classifies Hsp70s into four lineages: cytosolic A, cytosolic B, endoplasmic reticulum (ER), and mitochondria (Yu et al. 2021). The aim of the present study is to further validate this novel classification by annotating Hsp70s of *Drosophila melanogaster* . Our phylogenetic analysis demonstrated that *D. melanogaster* Hsp70s can be reasonably classified using this approach, unveiling novel insights into the relationship of Hsp70 family member genes. Based on the result, we propose new aliases of *D. melanogaster* Hsp70 family members.

According to the FlyBase, *D. melanogaster* has 13 Hsp70 genes (FBgg0000497; HEAT SHOCK PROTEIN 70 CHAPERONES), including 7 genes annotated as *Hsc70* (Table 1). The gene name “Hsc70” is probably based on the pioneering studies in early 1980s that showed constitutive expression of these genes under heat stress (Craig et al. 1983; Ingolia and Craig 1982), but later studies have shown that all of these Hsc70 genes are actually induced in response to other stresses. The *D. melanogaster* Hsc70Cb gene was also found in NCBI and FlyBase, but this gene was not included in FBgg0000497. This gene was included in the FlyBase gene group FBgg0000498 (ATYPICAL HEAT SHOCK PROTEIN 70 CHAPERONES), in which the gene product is described as a co-chaperone of canonical Hsp70s. Moreover, this gene encoded a product of about 88 kDa, and the sequence showed low identity with other Hsp70s. The Hsc70-6 was not included in FBgg0000497 either; the sequence of this gene was absent in NCBI. We therefore used only the 13 *D. melanogaster* Hsp70 genes in the FBgg0000497 for the following analyses.

Table 1. *Drosophila* *melanogaster* Hsp70/Hsc70 genes in NCBI and FlyBase

**Table:** 

Gene	NCBI Gene ID	FlyBase ID	Proposed alias	References showing stress-induced expression of the gene; remarks for Hsc70-6 and Hsc70Cb genes
Hsp70Aa	48581	FBgn0013275	HSP70cA1	(Colinet et al. 2010)
Hsp70Ab	44920	FBgn0013276	HSP70cA2	(Ogura et al. 2009)
Hsp70Ba	44921	FBgn0013277	HSP70cA3	(Sadanandan 2016)
Hsp70Bb	48582	FBgn0013278	HSP70cA4	(Chen et al. 2015)
Hsp70Bbb	50022	FBgn0051354	HSP70cA5	(Azad et al. 2009)
Hsp70Bc	48583	FBgn0013279	HSP70cA6	(Chen et al. 2015)
HSP68	42852	FBgn0001230	HSP70cA7	(Chen et al. 2015; Colinet et al. 2010; Ogura et al. 2009)
CG7182	38944	FBgn0035878	Not applicable	(Rehwinkel et al. 2004)
Hsc70-1	39542	FBgn0001216	HSP70cB1	(Chen et al. 2015; Štětina et al. 2015)
Hsc70-2	41609	FBgn0001217	HSP70cA8	(Chen et al. 2015)
Hsc70-3	32133	FBgn0001218	HSP70er1	(Štětina et al. 2015)
Hsc70-4	41840	FBgn0266599	HSP70cB2	(Štětina et al. 2015)
Hsc70-5	36583	FBgn0001220	HSP70m1	(Chen et al. 2015; Štětina et al. 2015)
Hsc70-6	5656927	FBgn0001221	Not applicable	Not included in Hsp70 (FBgg0000497) in FlyBase; Unannotated; Sequence not available in NCBI
Hsc70Cb	39557	FBgn0026418	Not applicable	Not included in Hsp70 (FBgg0000497) in FlyBase; a co-chaperon of canonical Hsp70 (FBgg0000498)

In the Bayesian phylogenetic tree, *D. melanogaster* Hsp70s were separated into cytosolic A, cytosolic B, ER, and mitochondrial linages (Fig. 1A). Yeast and cnidarian Hsp70s were basal to the *D. melanogaster* Hsp70s in each of cytosolic, ER, and mitochondrial lineages, indicating that the separation of these lineages were ancient. Importantly, Hsc70 genes did not form a cluster as expected. The Hsc70-2 gene belonged to the cytosolic A lineage with other Hsp70 genes; Hsc70-1 and Hsc70-4 genes were in the cytosolic B lineage; Hsc70-3 and Hsc70-5 belonged to the ER- and Mt lineages, respectively. To confirm the robustness of this phylogenetic analysis, we constructed a total of six phylogenetic trees combining two tree-building methods (Bayesian and maximum-likelihood) and three alignment methods (Clustal Omega, MUSCLE, and M-Coffee). All phylogenetic trees confirmed this result.

Detailed sequence inspections further found Hsp70 signature motifs (Yu et al. 2021) that supported the phylogenetic analysis. First, the lineage-specific motifs were well conserved in all *D. melanogaster* Hsp70 family members analyzed (Fig. 1B). Second, the extra serine residue known to be present in the ATPase domain of cytosolic A but not B lineage was found only in the cytosolic A Hsp70s of *D. melanogaster* (Fig. 1C). Third, the Hsc70-3 in the ER lineage had the KDEL motif at the C-terminal. Overall, these results showed that the phylogenetic approach can classify *D. melanogaster* Hsp70s in a consistent way with those for the entire metazoan spectrum (Yu et al. 2021) and for the rotifer *Brachionus plicatilis* sensu stricto, an emerging invertebrate model for evolutionary genetics (Grewal et al. 2022).

A limitation of the present study is that our phylogenetic analysis may not exactly represent the divergence time of closely related genes such Hsp70Aa and Hsp70Ab (Fig. 1A). Some Hsp70 genes are known to have been homogenized by gene conversion in *D. melanogaster* (Bettencourt and Feder 2002), as also reported for nematode (Nikolaidis and Nei 2004) and human (Kudla et al. 2004), and thus the genetic distance between some Hsp70 genes may not be proportional to the divergence time. However, this does not affect the conclusion of the present study. The gene conversion homogenizes the sequence within a species, possibly causing an underestimation of divergence time between isoform genes, but unlikely affects the identification of the ancient split of the four Hsp70 lineages. In addition, the nomenclature based on the existing gene sequences should be more robust than that based on the divergence time, which may be able to be better inferred by the future development of phylogenetic methods. Another limitation is the potential inconsistency between the phylogenetic classification and subcellular localization. For example, we cannot rule out the possibility that one may identify an ER-localized Hsp70 evolved from the cytosolic Hsp70 in future. However, the presence of such Hsp70 does not affect the current phylogenetic classification. Considering that the current classification based on phylogeny and organelle localization motifs can be applied to all tested metazoan Hsp70s, including those found by the genome-wide screening in human, *B. plicatilis* , and *D. melanogaster* , it would be beneficial to include the lineage in Hsp70 gene names.

The traditional names of *D. melanogaster* Hsp70 family members are still widely used, and it may not be a realistic idea to completely replace the gene names based on our phylogenetic analysis. However, we propose to add the Hsp70 alias in Table 1 for better understanding of their evolutionary relationship. Future studies with phylogenetic and evolutionary insights (e.g., those including cross-species sequence comparison) are encouraged to use these names that have incorporated the evolutionary information, avoiding the use of “Hsc70” that will bring unnecessary confusion.

## Methods

Sequences were obtained from the NCBI in January 2022. FlyBase IDs were retrieved from FlyBase version FB2022_01 (Attrill et al. 2016). Sequence alignment was performed using Clustal Omega (clustalo v1.2.4) (Sievers et al. 2011), M-Coffee (Wallace et al. 2006), and MUSCLE v3.8.1551. Both Bayesian and maximum-likelihood phylogenetic trees were constructed for the three different alignments. Best-fit models were determined for each tree by ProtTest-3.4.2 (Darriba et al. 2011) using the Bayesian information criterion. Bayesian trees were constructed using MrBayes 3.2.7a (Ronquist et al. 2012) with two independent runs, each of which had 100,000 generations. Every 10th tree was sampled, and burn-in was set to 25%. Maximum-likelihood trees were constructed using MEGA11 with 1000 bootstrap replications. Phylogenetic trees were visualized with FigTree (v1.4.4).
